# Quercetin conjugated with superparamagnetic iron oxide nanoparticles improves learning and memory better than free quercetin via interacting with proteins involved in LTP

**DOI:** 10.1038/s41598-019-43345-w

**Published:** 2019-05-03

**Authors:** Elnaz Amanzadeh, Abolghasem Esmaeili, Rezvan Enteshari Najaf Abadi, Nasrin Kazemipour, Zari Pahlevanneshan, Siamak Beheshti

**Affiliations:** 1 Department of Biology, Faculty of Sciences, University of Isfahan, Isfahan, Iran; 2 Department of Basic Sciences, School of Veterinary Medicine, Shiraz University, Shiraz, Iran; 3 Department of Chemistry, Catalysis Division, University of Isfahan, Isfahan, Iran

**Keywords:** Biochemical reaction networks, Spatial memory

## Abstract

Biomedical application of quercetin (QT) as an effective flavonoid has limitations due to its low bioavailability. Superparamagnetic iron oxide nanoparticle (SPION) is a novel drug delivery system that enhances the bioavailability of quercetin. The effect of short time usage of quercetin on learning and memory function and its signaling pathways in the healthy rat is not well understood. The aim of this study was to investigate the effect of free quercetin and in conjugation with SPION on learning and memory in healthy rats and to find quercetin target proteins involved in learning and memory using Morris water maze (MWM) and computational methods respectively. Results of MWM show an improvement in learning and memory of rats treated with either quercetin or QT-SPION. Better learning and memory functions using QT-SPION reveal increased bioavailability of quercetin. Comparative molecular docking studies show the better binding affinity of quercetin to RSK2, MSK1, CytC, Cdc42, Apaf1, FADD, CRK proteins. Quercetin in comparison to specific inhibitors of each protein also demonstrates a better QT binding affinity. This suggests that quercetin binds to proteins leading to prevent neural cell apoptosis and improves learning and memory. Therefore, SPIONs could increase the bioavailability of quercetin and by this way improve learning and memory.

## Introduction

Long-Term Potentiation (LTP) is a cellular mechanism by which synaptic plasticity of neural cells is strengthened, resulting in the preservation of memories. Learning and memory are among the most important cognitive functions, which are gradually lost with aging. Learning and memory are mainly dependent on the synaptic plasticity by which long-term signal transduction leads to strengthening the synapses between neurons. This process is referred to LTP^1^ while it is affected by several factors such as high blood fat, oxidative stress, etc^2–4^. Therefore, in order to prevent memory impairment and the development of neurodegenerative diseases, maintenance of LTP seems necessary^5^. Neurotrophin signaling pathway is closely related to the LTP so that induction of LTP leads to organization of new signal transductions between neurons and inhibit memory impairment and neurodegeneration^6,7^. On the other hand, improvement of learning and memory can also be due to the prevention of apoptosis. Consistency, it has also been shown that prevention of neurodegenerative diseases in addition to inhibition of neural cell apoptosis, restores LTP^8,9^.

**Figure 1:**
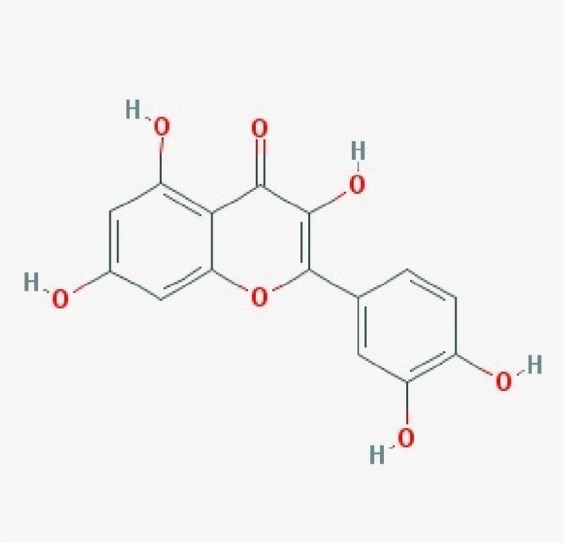
2D structure of QT obtained from PubChem^120^.

The biological activities of flavonoids in the central nervous system (CNS) are due to their abilities to protect susceptible neurons, increasing the function of existing neurons, stimulating neuronal regeneration, and induction of neurogenesis^10–13^. Quercetin (QT) (2-(3,4-dihydroxyphenyl)-3,5,7-trihydroxy-4H-chromen-4-one, Fig. 1) is one of the most abundant flavonoid compounds in the daily diet which has been known to protect neurons against the oxidative stress and apoptosis^14^. It is proposed that quercetin has inhibitory effects on proteins while this feature hasn’t been studied sufficiently. However, the major drawback of QT *in vivo* treatment is its low bioavailability so that it shows no significant effects on the induction of LTP^15^.

Interaction of QT with proteins involved in various signaling pathways has been reported previously^16^. In this regard, it has also been revealed that QT leads to decrease in cell apoptosis in the hippocampus which is the center of processing the spatial memory and this property can be considered as a preventive treatment against oxidative stress^14,17^. Besides, the exact molecular mechanism of QT action in the neural cells has not been revealed so far; hence, we decided to find cellular targets of QT through which QT treatment leads to the improvement of the learning and memory using bioinformatics tools. For this purpose, in addition to the use of experimental tests in order to confirm the beneficial effect of QT on learning and memory when there is no oxidant factor, docking software including *Hex*, *AutoDock*, and *LigandScout* were used to find interactions of QT with the proteins of considered signaling pathways. QT docking sites have been studied in some cases such as inhibitory effect on ATPase of sarcoplasmic reticulum^18^, protein disulfide isomerase^19^, inhibition of glucose efflux via binding to CLUT1^20^, inhibition of Akt activity leading to the cell survival^21^, inhibition of Cs^2+^- ATPase^22^, etc. However, there is no clear finding of specific targets of quercetin within the cell. As the first study with this aim, we decided to assess interactions of quercetin with all proteins in neurotrophin and LTP signaling pathways computationally. First, we should be sure about the beneficial effects of quercetin on learning and memory in healthy organisms. Therefore, intact rats were used to assess the effect of QT on learning and memory in the absence of any oxidative agent through which the main beneficial effect of QT will be dependent on the interactions of QT with proteins. In addition, since quercetin has low bioavailability, we also assessed the efficiency of a delivery system, which had proposed previously on the enhancement of its bioavailability. A number of methods have been proposed in order to increase quercetins bioavailability.

The use of quercetin derivatives such as quercetin aglycones^23^, emulsifiers^24,25^, conjugates^26–28^ have been proposed which showed satisfactory results in terms of accessibility and bioavailability. A novel method for delivering therapeutic compounds is the use of nanotechnology. Manufacturing quercetin included nanostructures including conjugates^29–31^, nanotubes^32^ have been conducted in order to increase quercetin bioavailability and its solubility. However, there are few studies related to the use of quercetin-included nanoparticles in order to deliver this compound to brain tissue.

Nanoparticles (NPs) are important because of their unique properties such as high surface to mass ratio, ability to absorb, and also carry other compounds that leads NPs to be effective for carrying drugs, proteins, and probes^33^. However, NPs have also limited rate of passage from the blood-brain barrier (BBB)^34^. In this regard, nose to brain method has been studied in order to increase drug concentrations in brain tissue and prevent drug disruption in the gastrointestinal tract. This method has been introduced with a high success rate in drug delivery to the brain while the main disadvantage of this route is low permeation of drug compounds^35,36^. On the other hand, the use of nanoparticles helped to overcome drug resistance in some diseases in which therapeutic compounds could not transverse across the barriers such as the blood-brain barrier (BBB). Superparamagnetic iron oxide nanoparticles (SPION) have attracted a lot of attention in biomedical applications. SPION has unique properties including a high ratio of spin polarization and high conductivity and especially a superparamagnetic nature^37^. SPION as a carrier system has many advantages over other nanoparticle carrier systems. Iron oxide NP is biocompatible, biodegradable, and superparamagnetic and therefore controllable by an external magnetic field. This nanoparticle with different coatings has various biological applications including highly sensitive diagnostic tests, drug delivery, gene delivery, magnetic resonance imaging, cell tracking, tissue engineering, magnetic hyperthermia, magnetic resonance imaging (MRI), thermal therapy, targeting amyloid beta (Aβ) in the brain arteries, inhibiting the microglial cells, and DNA detection^38–41^. However, potentially they may be toxic and could destruct the functions of the main organelles and macromolecules of the cell, such as DNA, nucleus, and mitochondria^42,43^. The toxicity of SPIONs depends on important factors such as the size of the nanoparticles, the exposure time, the type of NP coating, and the type of tissue^44^. Our previous study showed that dextran-coated iron oxide nanoparticles have a minor cell toxicity at high dosage^44^. In another study, we showed that SPIONs could enhance the biodistribution of quercetin into the brain^45^. Small size, low toxicity, and targeted drug delivery possibility via magnetic fields are among the most important advantages for the SPION population^46,47^. We used quercetin-SIOPN conjugation system in order to enhance bioavailability of quercetin, which can also help to its inhibitory effects on proteins, especially in the brain. QT-SPION (QT-Fe_3_O_4_) has been used in order to improve learning and memory in rats with memory impairment^48^. Additionally, the enhanced bioavailability of this delivery system has been shown by Najafabadi *et al*.^45^. However, there is no report about its effects on learning and memory in intact rats.

Hereby, we studied the effect of QT and QT-SPION on learning and memory of intact rats in order to confirm beneficial effects of QT and designed delivery system on the improvement of learning and memory. In order to find specific targets of inhibitory activity of quercetin, a comprehensive screening was also conducted as the primary step for demonstrating potential inhibitory effects of quercetin on proteins in signaling pathways related to learning and memory.

## Results

### Characterization of QT- SPIONs

**Figure 2:**
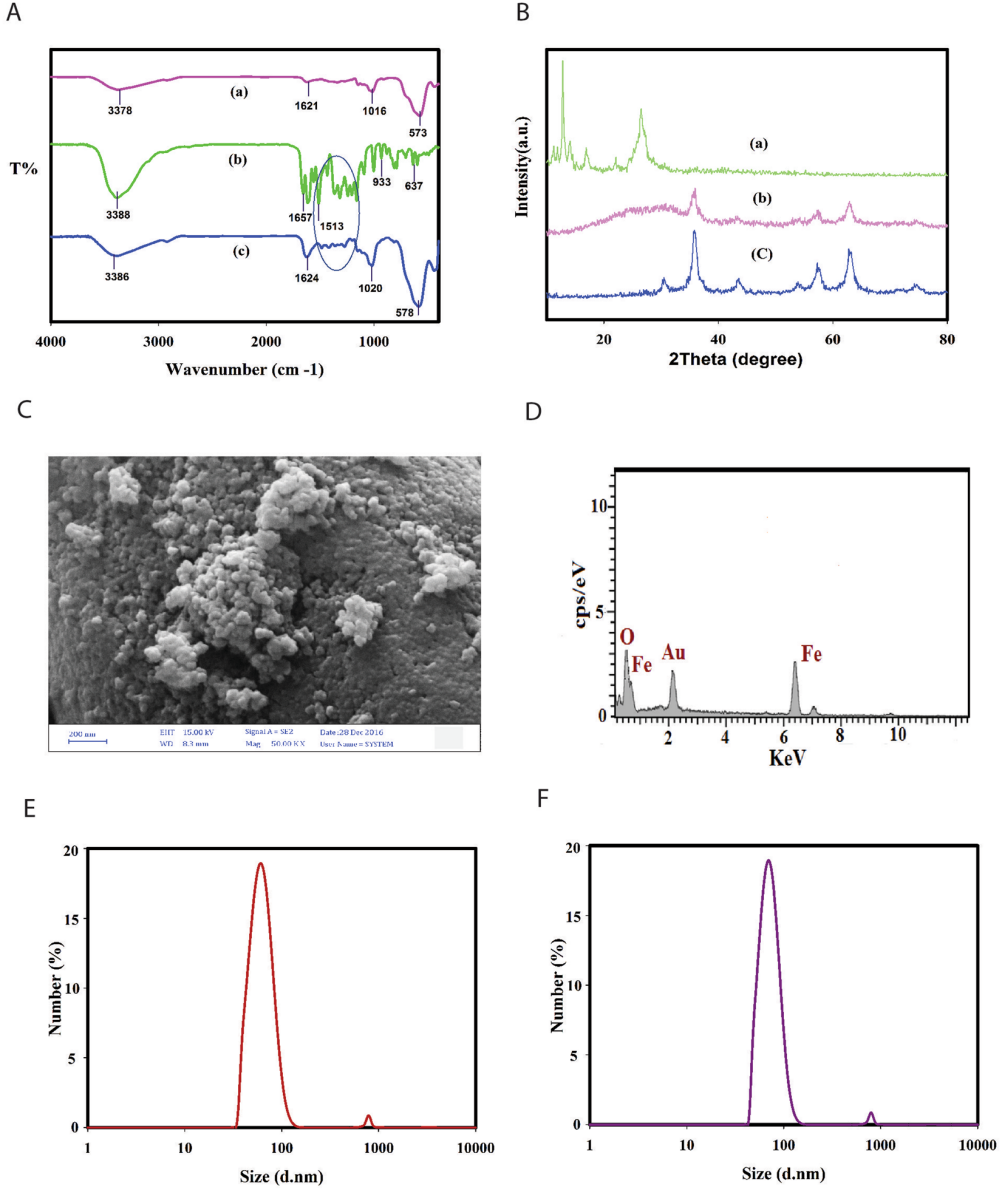
Characterization of QT-SPION. (**A**) FT-IR spectra for the (a) dextran coated, (b) pure QT and (c) QT conjugated SPIONs. (**B**) XRD pattern for the (a) QT, (b) dextran coated SPIONs and (c) QT conjugated SPIONs. (**C**) SEM image of QT conjugated SPIONs. (**D**) SEM-EDX spectrum of QT conjugated SPIONs. SPION: superparamagnetic iron oxide nanoparticle. (**E**,**F**) Dynamic light-scattering (DLS) spectra of dextran coated SPIONs and QT-SPIONs dispersions.

The FTIR spectrum of dextran-coated SPIONs, QT and QT conjugated superparamagnetic nanoparticles (QT-SPION) are shown in Fig. 2A. The spectrum of dextran-coated SPIONs showed the broad peaks at 3378 cm^−1^ represent the OH stretching vibration of the hydroxyl groups and a strong absorption band at 573 cm^−1^ which was assigned to the Fe–O vibration frequency of magnetite spinel structure in Fig. 2A(a). The FTIR of quercetin showed characteristic bands corresponding to C=O absorption at 1657 cm^−1^, OH groups at 3388 cm^−1^, and a region corresponding to C-O stretching at 1150–1070 cm^−1^. In addition, the peak 933 cm^−1^ represents C-H bending vibration of aromatic groups in Fig. 2A(b). The spectrum of the QT-SPION showed broad absorption bands at 3386 cm^−1^, which represented the stretching vibrations of hydroxyl groups and other characteristic bands, that confirmed the successful conjugation of quercetin on the superparamagnetic nanoparticles in Fig. 2A(c). The XRD measurements were performed to identify the crystal phases present in the samples. Figure 2B(a–c) shows the powder XRD pattern of QT, dextran coated SPIONs and QT conjugated superparamagnetic nanoparticles. This reveals a pattern similar to the crystalline magnetite Fe_3_O_4_ and showed all the major corresponding peaks at 2Ѳ = 30.1°, 35.4°, 43.9°, 53.4°, 57.0° and 62.6° confirming the presence of the crystalline structure of the magnetite. The XRD pattern of pure QT shows numerous distinct peaks characteristic of high crystalline nature^49^, but no diffraction peaks were observed in QT-SPIONs compared to pure QT. This finding provided evidence that free QT in the QT-SPIONs was indeed converted from a crystal to an amorphous state^50^. The exterior morphology of the QT conjugated SPIONs was pictured by FE-SEM data demonstrated that quercetin conjugated NPs show a spherical shape (Fig. 2C). Dynamic light scattering (DLS) was used to measure the hydrodynamic size of magnetite nanoparticles. As shown in Fig. 2(E,F), the size distribution diagram of the dextran coated SPIONs in aqueous solution displays a uniform distribution with an average hydrodynamic diameter of 60 nm that increase to 69.5 nm for the QT-SPIONs due to the conjugation of quercetin to the dextran coated SPIONs.

### Morris water maze (MWM) test

**Figure 3:**
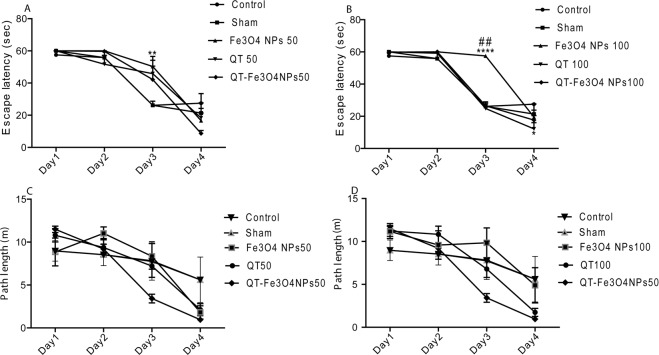
Effect of treatment with QT alone or in conjugated with SPION (7 days) on the performance of spatial memory acquisition phase in Morris water maze. Mean escape latency (**A**,**B**) and path length (**C**,**D**) across all training days. As shown the mean latency to find the platform declined progressively in all animals. Data expressed as mean ± S.E.M. of 6 animals per group. Sham, Vehicle of QT; QT; SPIONs, superparamagnetic iron oxide nanoparticle; QT-SPIONs; QT conjugated with superparamagnetic iron oxide nanoparticle; 50, 50 mg/kg; 100, 100 mg/kg. *P < 0.05, **P < 0.01 and ****P < 0.0001 versus control and sham groups. ^##^P < 0.01 versus other groups.

The mean escape latency of each training day was calculated using data from four training rounds per day. The mean escape latency of all groups was compared using a one-way ANOVA test while no significant difference was observed (P = 0.4848, Fig. 3A,B). Similar results were obtained for the second training day. However, on the third training day, SPION (50 mg/kg and 100 mg/kg) groups demonstrated significantly higher escape latency compared to control and sham groups (P < 0.01, and p < 0.001 respectively). In addition, two-way ANOVA test results showed that during 5 training days, QT and QT-SPIONs treatment led to shorter escape latency compared to the control group. In contrast, other groups demonstrated no significant differences in comparison to the control group (Fig. 3B). QT with concentrations of 50 mg/kg (p < 0.05) and 100 mg/kg (p < 0.001), and QT- SPION (50 mg/kg) revealed significantly shorter escape latency during the training days (p < 0.05) compared to the control group while the other groups demonstrated no significant differences in comparison with control group. The other parameter assessed in this study is path length (animal total distance traveled) which was obtained in training days (Fig. 3C,D). According to the results, the path length in all groups was shortened during the training period. Two-way ANOVA results showed no significant differences between the studied groups.

**Figure 4:**
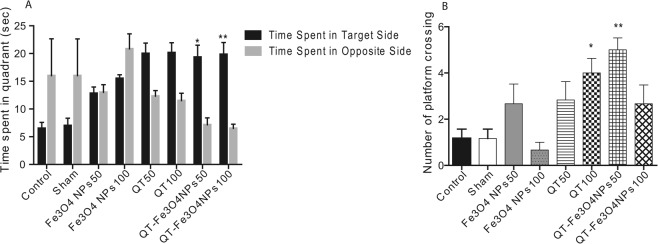
Effects of QT on spatial learning and memory in the rat. (**A**) Effect of QT treatments on learning ability in terms of total time spent in the platform quadrant (MWM); (**B**) effect of QT treatments on memory index in terms of number of platform crossings. Values are represented as mean ± SEM (n = 6). Statistical significance was determined using two-way ANOVA (followed by tukey,s multiple comparison test) at *P < 0.05, and **P < 0.01.

The third investigated parameter measured at probe trial day is time spent in the target zone, while the target zone is the zone containing plate during the training days. Time spent in the target zone (in the present study, Z3 zone was considered as the target zone) was compared to time spent in the opposite zone (In the present study, Z1 zone). According to the results, QT-SPION-50 and QT-SPION-100 mg/kg groups demonstrated significantly longer time spent in the target zone compared to other groups (p < 0.05 and p < 0.01 respectively, Fig. 4A).

Additionally, the number of plate crosses of the studied groups was recorded in the probe trial day (6^th^ day). The obtained results revealed that number of plate crosses in rats treated with QT (100 mg/kg, p < 0.05), and QT- SPIONs (50 mg/kg, p < 0.01) was significantly more than the other groups plus comparison of this parameter between other studied groups showed no statistically significant difference (Fig. 4B).

#### Histological analysis and quantitative tissue iron measurement

**Figure 5:**
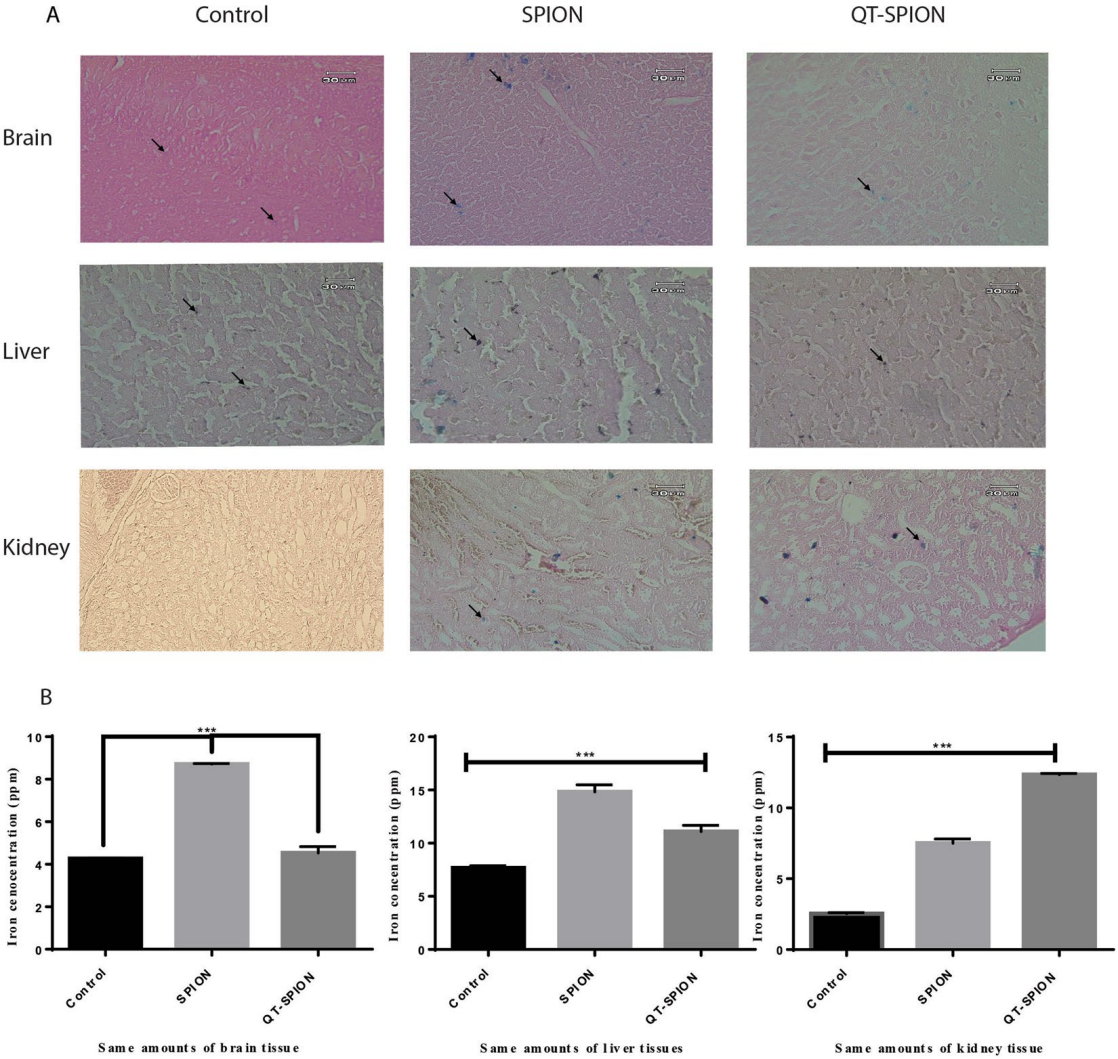
Results of Prussian blue staining and ICP-MS. (**A**) Prussian blue staining of brain, liver and kidney tissues of control group, SPION group and QT-SPION group. (**B**) Results of one-way ANOVA analysis of ICP results obtained for brain, liver and kidney tissues. ***p-value < 0.001.

Prussian blue staining results showed that iron concentration is not significant in the brain tissues of the control group (Fig. 5A). However, iron aggregations in brain tissues of groups treated with SPION and QT-SPION were observable (Fig. 5A). Prussian blue staining on kidney and liver tissues of control, SPION, and QT-SPION groups were also performed and the obtained results revealed that more iron aggregations are found in the liver of groups treated with SPION and QT-SPION compared to the control group (Fig. 5A). The results obtained for kidney tissues also showed similar results with liver, so that iron aggregations were observed more in SPION and QT-SPION treated groups than the control group (Fig. 5A)

These results were consistent with the results obtained from ICP analysis. It was observed that iron concentration in brain tissue of groups treated with SPION was significantly higher than groups treated with QT-SPION and control group (P < 0.001) (Fig. 5B). In addition, the concentration of iron in liver tissue of QT and QT-SPION groups was higher than the control group, however, the difference was not significant between control and QT-SPION groups (Fig. 5B). In contradiction with other results, concentrations of iron were significantly higher in QT-SPION group compared to the SPION and control groups (Fig. 5B).

### Evaluation of QT effects on proteins involved in signaling pathways of neural cells using bioinformatics tools

Hex software was used to calculate free energy between the proteins of the prepared database and QT and SPIONs as distinct ligands. Interaction energies of QT and SPIONs with all of the proteins in the database were calculated and recorded in Table 1. The obtained energies from the interactions between SPIONs and QT with proteins were compared using a one-way ANOVA test. The results revealed a significant difference between two groups (P = 0.000). Accordingly, SPIONs cannot be considered as a ligand compared to the QT.

**Table 1:** List of proteins involved in the apoptosis, long-term potentiation (LTP), and neurotrophin signaling pathways and free energies of their interactions with QT obtained using Hex software.

Apoptosis				QT		Fe3O4		PubChem ID of Inhibitor	Inhibitor	
		KEGG entry	PDB ID	Hex	Autodock	Hex	Autodock		Hex	AutoDock
1	BID	K04726	**5UA4**	−216.3	−7.69	−103.52	−1.58	11547341	−289.23	−7.86
2	BCL2	K02161	**5B1Z**	−278.6	−8.41	−98.75	−1.57	49846579	−289.37	−9.39
3	BAX	K02159	**1F16**	−274.4	−7.46	−102.01	−1.81	10129115	−301.46	−9.42
4	SMAC	K10522	**1few**	−108.283	−0.18	−87.39	0.25	11200722	−256.89	−8.75
5	HTRA2	K08669	**1LCY**	−266.1	−7.81	−97.43	−2.21	1102771	−301.89	−9.03
6	Cyc C	K08738	**2N3Y**	−285.5	−8.38	−100.04	−2.36	4100	−187.61	−5.86
7	FADD	K02373	**1a1w**	−243.7	−7.76	−115.77	−2.21	5351169	−223.21	−5.85
8	TNFR1	K03158	**1f2h**	−281.2	−8.74	−104.47	−2.17	47205810	−262.34	−7.01
9	APAF1	K02084	**5wvE**	−256.79	−10.07	−99.27	−1.92	25108684	−233.62	−3.45
10	Caspase8	K04398	**3h11**	−275.2	−8.31	−116.87	−1.67	5497174	−361.31	−10.56
11	Caspase9	K04399	**3v3k**	−293.6	−8.31	−75.23	−2.40	10032582	−303.29	−10.58
12	Cas3	K02187	**1gfw**	−251.2	−8.36	−117.37	−2.45	16760394	−268.96	−9.01
**Neurotrophin Signaling Pathway**										
13	NGFB	K02582	**4edw**	−113.4	−0.11	−98.14	−1.77	9943465	−292.38	−8.46
14	BDNF	K04355	**1b8m**	−226.9	−6.33	−77.57	−1.88	11612445	−275.43	−8.04
15	NTF3	K04356	**1b8k**	−253.0	−7.48	−87.17	−1.47	10074640	−268.73	−8.21
16	NTRK1	K03176	**5km1**	−282.6	−8.86	−112.74	−2.17	59397065	−296.37	−9.08
17	NGFR	K02583	**2n80**	−272.8	−7.73	−104.17	−2.14	17756791	−307.59	−7.98
18	SORT1	K12388	**4po7**	−282.86	−10.00	−96.70	−1.64	1061996	−261.83	−6.77
**MAPK pathway**										
19	FRS2	K12461	**2mfq**	−260.01	−7.55	−95.06	−1.14	57379345	−280.91	−8.30
20	CRK	K04438	**1ju5**	−273.2	−8.47	−96.06	−2.05	9549301	−266.97	−7.46
21	BRAF	K04365	**5j17**	−260.8	−8.14	−118.70	−2.03	44223999	−298.03	−10.56
22	MEKK3	K04421	**4y5o**	−256.8	−7.41	−123.49	−1.86	11544170	−268.39	−8.40
23	MEK5	K04463	**2o2v**	−248.2	−6.97	−103.70	−1.80	1530	−279.62	−9.06
24	MAPK7	K04464	**4zsg**	−269.0	−8.02	−93.29	−2.41	747994	−297.05	−9.08
25	ATF4	K04374	**1ci6**	−234.6	−6.67	−116.89	−1.42	AID 1060412	−298.30	−10.69
26	MSK1	K04445	**3kn5**	−310.29	−10.36	−89.65	−2.35	71584481	−257.36	−7.78
27	GRB2	K04364	**5gjh**	−299.9	−8.21	−109.740	−2.23	3035817	−322.49	−9.68
28	SOS	K03099	**4ruv**	−235.8	−7.83	−122.086	−2.19	374536	−257.39	−8.49
29	HRAS	K02833	**4uru**	−256.44	−9.46	−111.957	−2.05	159324	−279.23	−10.08
30	MEK1	K04368	**5eym**	−260.39	−9.79	−98.10	−1.90	389898	−273.48	−9.93
31	ABL	K06619	**5hu9**	−247.69	−10.18	−100.02	−2.16	3320547	−258.53	−10.71
32	SHC1	K06279	**4jmh**	−236.2	−8.11	−103.428	−2.15	49806720	−279.23	−9.02
33	IRS1	K1672	**3bu3**	−246.36	−9.15	−110.721	−1.95	65110	−279.33	−9.28
34	PI3K	K02694	**4ovu**	−234.4	−9.03	−121.153	−1.68	6918289	−258.19	−9.58
35	PDPK1	K06276	**5lvl**	−276.08	−10.24	−124.237	−1.95	10027278	−288.54	−10.98
36	Akt	K04456	**5kcv**	−281.58	−9.19	−113.825	−2.01	5329102	−286.31	−8.98
37	NFKBIB	K02581	**4kik**	−275.3	−8.20	−10.718	−1.93	9549298	−285.47	−8.45
38	NFKB1	K02580	**1NF**	−261.3	−8.33	−106.415	−1.46	5353432	−296.37	−8.95
39	FOXO3	K09408	**2k86**	−255.6	−7.60	−130.845	−1.88	122382714	−266.18	−7.83
40	GSK3B	K03083	**5k5n**	−289.8	−8.32	−121.718	−2.08	16760703	−291.17	−8.35
41	PLCG1	K01116	**5eg3**	−246.2	−7.37	−124.119	−2.11	4784	−253.34	−7.56
42	PRKCD	K06068	**3uej**	−245.27	−9.20	−113.56	−1.85	59783146	−283.49	−10.56
43	CAMK2	K04515	**5ig3**	−235.6	−7.81	−97.41	−1.57	5312122	−267.13	−9.23
44	TNFSF6	K04389	**5l19**	−268.0	−7.59	−128.295	−2.61	9831419	−276.39	−8.95
45	TRAF6	K03175	**4z8m**	−230.2	−7.95	−129.492	−1.96	1061998	−267.43	−8.22
46	Cdc42	K04393	**2kb0**	−280.1	−9.33	−120.023	−2.02	2950007	−261.09	−8.80
47	Rac1	K04392	**4uip**	−265.5	−8.23	−127.117	−2.49	16759159	−266.69	−10.02
48	RIPK2	K08846	**5j79**	−196.4	−9.38	−119.713	−2.38	56602558	−267.68	−9.47
49	IRAK1	K04730	**5kx7**	−236.5	−8.75	−120.679	−1.97	11983295	−283.16	−10.27
50	JNK	K04440	**4E73**	−250.41	−7.98	−142.393	−1.84	8515	−273.12	−9.13
51	TP73	K10148	**2nb1**	−263.47	−7.66	−119.489	−1.86	4817	−275.63	−7.73
52	TP53	K04451	**3HF1**	−278.73	−8.51	−121.457	−1.62	3213	−254.49	−7.88
53	CREB	K04374	**1DH3**	−234.74	−6.73	−114.903	−1.50	329397	−289.36	−8.06
54	CBP	K04498	**4ouf**	−250.22	−8.01	−125.641	−2.03	1285941	−263.45	−8.14
55	RSK2	K04373	**4NUS**	−301.65	−8.38	−111.789	−1.73	25023738	−293.91	−7.13

Docking results obtained from Hex software for QT and the proteins of the prepared database showed that the lowest interaction energy was obtained by interacting with MSK1 as −310 Kcal/mol. Since the interaction energies obtained from the other proteins and QT had not significant difference with MSK1-Q, additionally due to the variation of results obtained in similar studies and lack of a standard to determine inhibitory effect of a chemical compound, and on the other hand the obtained energies were as low as appropriate to consider QT as an inhibitor, hence at the next step evaluation of interaction energy and docking were conducted using *AutoDock* (*AutoDock 4*.*2*.*6)* software. According to the results obtained from this software, Fe_3_O_4_ cannot also be considered as an inhibitor due to low interaction energies. MSK1-Q complex had also the lowest interaction energy while the same issue had not been solved. Due to the lack of significant differences between the interaction energies of QT and various proteins, it did not seem reasonable to introduce several proteins as candidates for inhibition by QT that leads to improvement of learning and memory. Meanwhile, there was consistency between the results obtained from *AutoDock* and *Hex* software. So that the difference of interaction energies between QT with each candidate protein was similar in results obtained from two software.

In order to obtain the most probable target for QT in the brain and mainly hippocampus that leads to improvement of mechanism learning and memory, we decided to compare interaction energies of QT and candidate proteins with interaction energies of the proteins and their specific inhibitors that have previously introduced as efficient inhibitors of each protein. Since the effect of inhibitors have been studied experimentally and they have been proved to be appropriate treatments against target proteins, the comparison of interaction energies between QT and specific inhibitor of each protein can help to find out whether QT inhibitory effect is significant and efficient or not. Accordingly, if interaction energy between QT and each protein was lower than the same index between the protein and its specific inhibitor and ki value of inhibitor was lower than QT, then QT can be considered as a better inhibitor for that protein. While if not, then QT isn’t considered as an effective inhibitor as the specific inhibitor and its effect can be ignored compared to the other proteins.

Accordingly, PubChem database, PubMed, and PDB database were searched in order to find the most effective inhibitors of proteins that had been used for the treatment of several diseases. Since many of the proteins of apoptosis signaling pathways are the targets of cancer therapy, several chemical compounds were found as inhibitors. In this study, we tried to evaluate interactions status of all the inhibitors while they may not include all the available inhibitors. Then inhibitors with the best docking results were reported to compare with QT. Table 1 also presents the inhibitors with the PubChem IDs. However, comparison of interaction energies of protein-inhibitor and protein- QT revealed that in certain cases, the calculated energy of protein-inhibitor was lower than protein- QT and therefore, in these cases, QT mainly is not considered as an inhibitor.

Results obtained for 8 proteins including *MSK1*, *RSK2*, *P53*, *Cdc42*, *CRK*, *FADD*, *Apaf1*, and *CytC* were also analyzed using *LigandScout* which investigates pharmacophore feature of QT and inhibitors of each protein and determines the number and types of the bounds. Since the viewpoint of this software is an evaluation of ligands as therapeutic compounds, it was important to recheck binding state of compounds.

**Figure 6:**
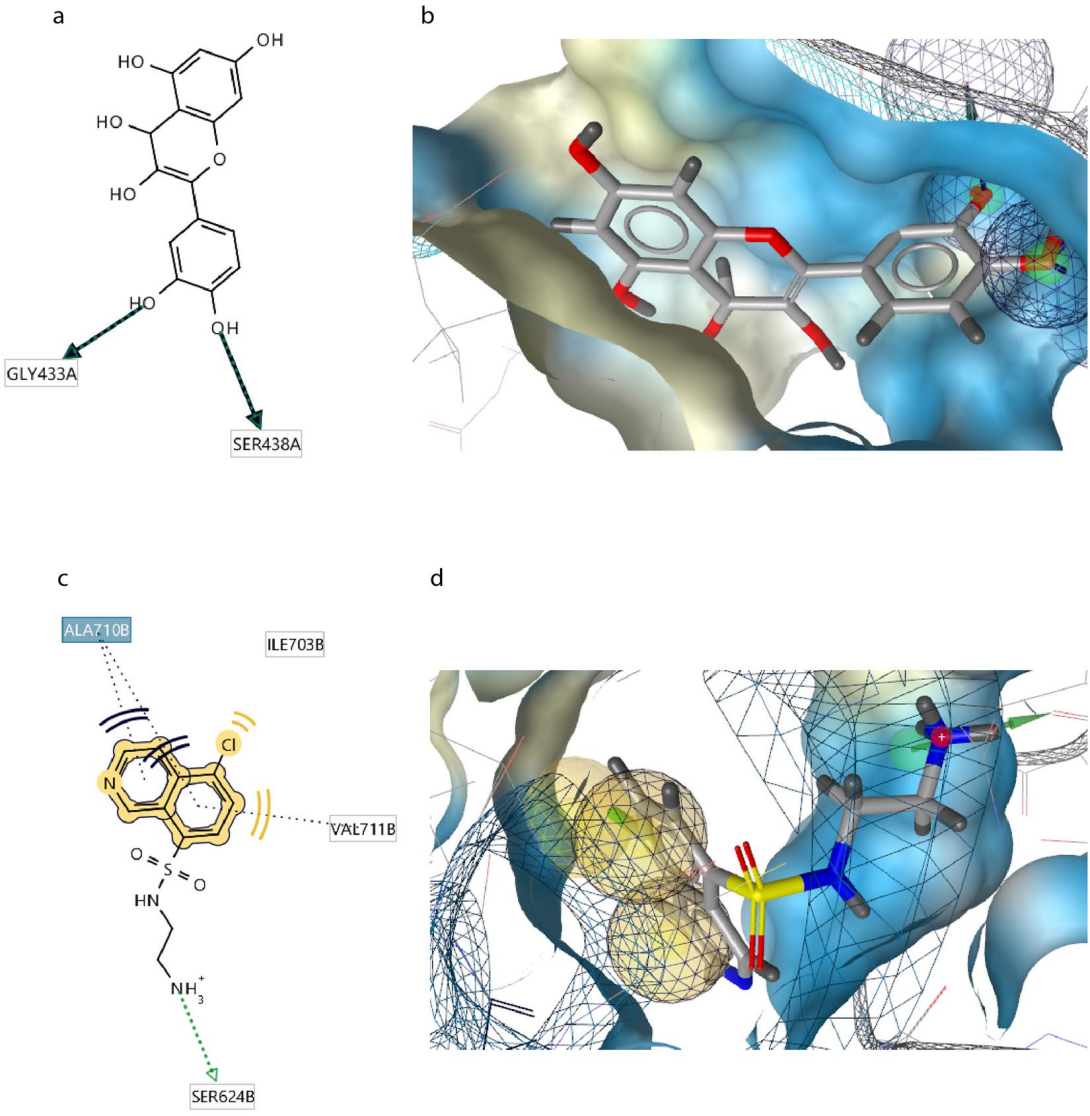
Schematic representations of interactions of Q-MSK1 and Inhibitor-MSK1 complexes. (**a**) Interactions of Q-MSK1 complex including three hydrogen bonds. (**b**) Interactions of Q-MSK1 complex including three hydrogen bonds displayed as (**d**). (**c**) Interactions of Inhibitor-MSK1 complex including one hydrogen bond and three hydrophobic interactions. (**d**) Fig. 5B: Interactions of Inhibitor-MSK1 complex including three hydrogen bonds displayed as 3D.

**Figure 7:**
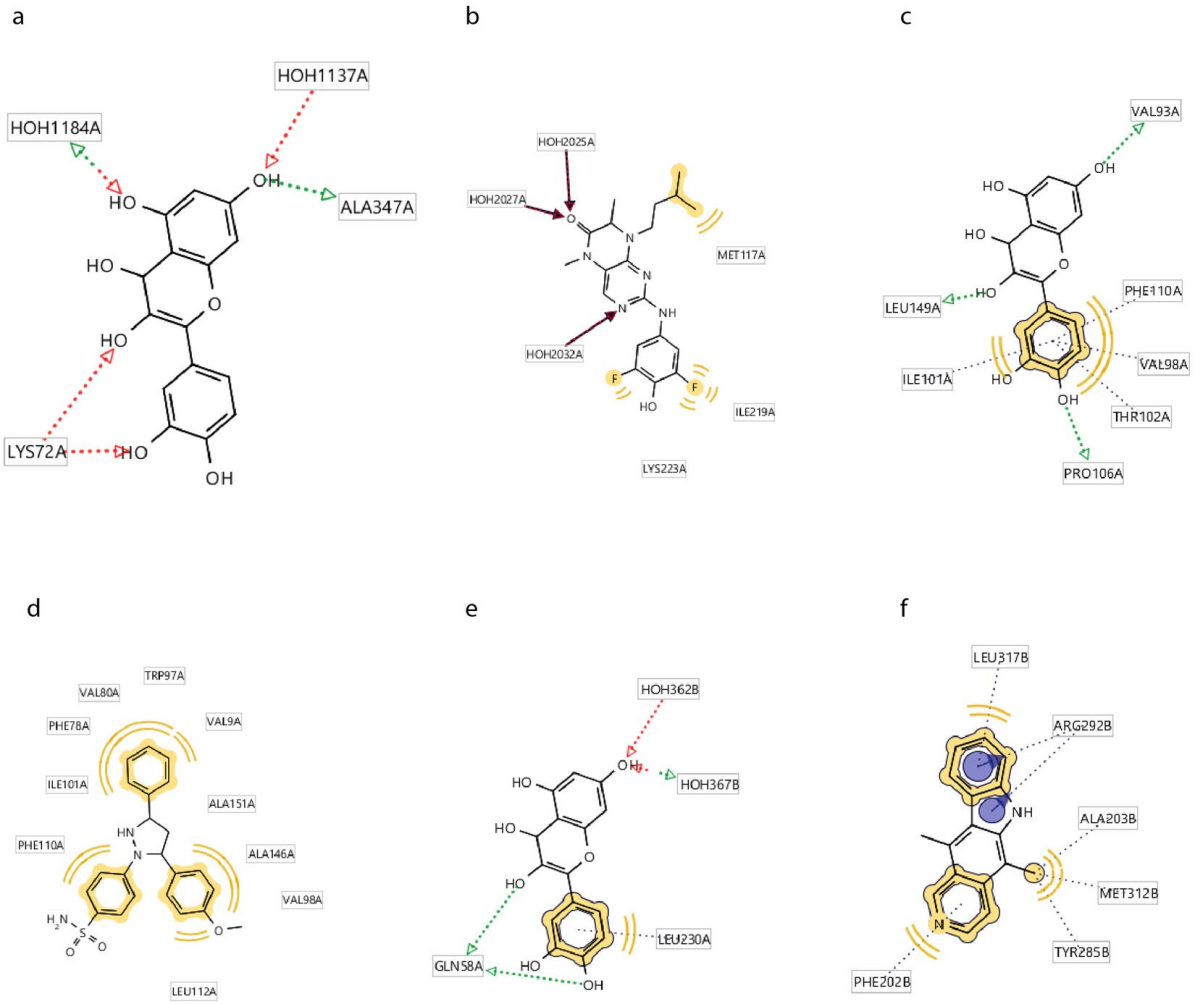
Interactions involved in (**a**) RSK2-Q, three hydrogen bonds) RSK2-Inhibitor, (**b**) three hydrogen bonds and two hydrophobic interactions, (**c**) Cdc42-Q, three hydrogen bonds, and four hydrophobic interactions, (**d**) Cdc42-Inhibitor, three hydrophobic interactions, (**e**) P53-Q, three hydrogen bonds, and (**f**) P53-Inhibitor, three hydrogen bonds.

The first protein with significantly lower interaction energy than protein-inhibitor was Mitogen Stress Kinase 1 which is a kinase and is found in several subcellular locations in various cells including neural cells of adult rats^51^. This kinase as a key member of MAPK signaling pathway plays an important role in phosphorylation and activation of several proteins which leads to the survival or death of the cells through neurotrophin or apoptosis signaling pathways^52^. After minimization of MMFF94 energy of interactions between ligand and side chains of complex obtained from docking analysis QT, as a pharmacophore, forms three hydrogen bonds with Gly433 and two hydrogen bonds with Ser438 at chain A which locate on protein kinase domain in a hydrophobic pocket consisting 38 amino acids mostly amino acids with hydrophobic side chains (Fig. 6a,b). This finding highlights the ability of QT to form various hydrogen bonds via its OH groups. In comparison, due to the results obtained from LiganScout, MSK1 and inhibitor can form a hydrogen bond with Ser624 on chain B of MSK1 and three hydrophobic interactions with Val 711, Ile 730, and Ala 710 on chain B which composes the ribosomal protein S6 Kinase domain (Fig. 6c,d). It can be observed that lower interaction energy obtained for QT-MSK1 interaction is due to the more hydrogen bonds and the number of amino acids involved in the interaction. In the present study, QT formed four hydrogen bonds with Lys72, and Ala374 amino acids of RSK2 that all involved in the protein kinase domain 1. In addition, comparison of RSK2-Q complex (Fig. 7a) with RSK2-inhibitor (Fig. 7b) complex revealed that QT is a more effective candidate to interact and inhibit this protein (Table 2).

**Table 2:** Amino acids and motifs interacted with the QT and ligand.

	QT		Inhibitor	
	H-Bond Energy	Domain	H-bond Energy	Domain
P53	−22.257	**Ribonucleoside-diphosphate reductase (ChainA- residues 37–48)**	−2.688	**Ribonucleoside-diphosphate reductase (ChainA- residues 96–228)**
Cdc42	−66.856	**Cell division control protein 42 homolog (Residues: 13–100- ChainA)**	−6.235	**Cell division control protein 42 homolog (Residues: 22–170- ChainA)**
MSK1	−7.926	**DNA-directed DNA polymerase (ChainA: 433–566)**	−14.871	**DNA-directed DNA polymerase (ChainA: 432–568)**
RSK2	−10.28		−28.37	
CRK	−38.926	**SH2 Domain(Residues: 70–113)**	−8.782	**SH2-SH3 Domain (Residues: 56–226)**
FADD	−23.646	**DED domain (Residues: 64–70)**	−16.26	**DED domain (Residues: 64–120)**
APAF1	−7.62	**Apoptotic protease-activating factor 1 (203–350)**	−21.39	**Apoptotic protease-activating factor 1 (203–300)**
CytC	−26.97	**CytC(53–103)**	−34.21	**CytC(65–103)**
Bax	−2.36	**BH3-BH2 Domain (Residues: 35–135)**	−13.47	**BH3-BH2 Domain (Residues: 35–160)**

Cell division cycle 42 is an important kinase and a key member of CamK||-Cdc42-PAK2 signaling cascade^27^. QT binds to this protein via three hydrogen bonds and one hydrophobic interaction in which amino acids of Val93, Val98, Ile101, Thr102, Pro106, Phe110, and Leu149 in the GTP nucleotide binding domain (Fig. 7c) contributed while inhibitor of this protein binds to the same domain via three hydrophobic interactions (Fig. 7d) which results in a significant difference in the interaction energies between two ligands and proves the more powerful interactions and inhibitory effect of QT.

P53- QT complex composes five hydrogen bonds and one hydrophobic interaction in which OH groups of QT and amino acids of P53 including Leu230 and Gln58 contribute (Fig. 7e). On the other hand, P53-inhibitor complex has been composed based on three hydrophobic interactions plus two interactions between aromatic ring of QT and amino acids including Leu137, Arg292, Ala2.3, Met312, Tyr285, and Phe282 (Fig. 7f). All of the amino acids contributing in both complexes locate on the ribonucleoside diphosphate reductase subunit; however, P53- QT interaction mainly composes of hydrogen bonds in contradiction with the P53-inhibitor complex which mainly composes of hydrophobic interactions and this difference is consistent with the difference in the interaction energies of two complexes. In addition, similar to kinases, QT also binds to the ribonucleoside binding domain of P53 (Table 2).

**Figure 8:**
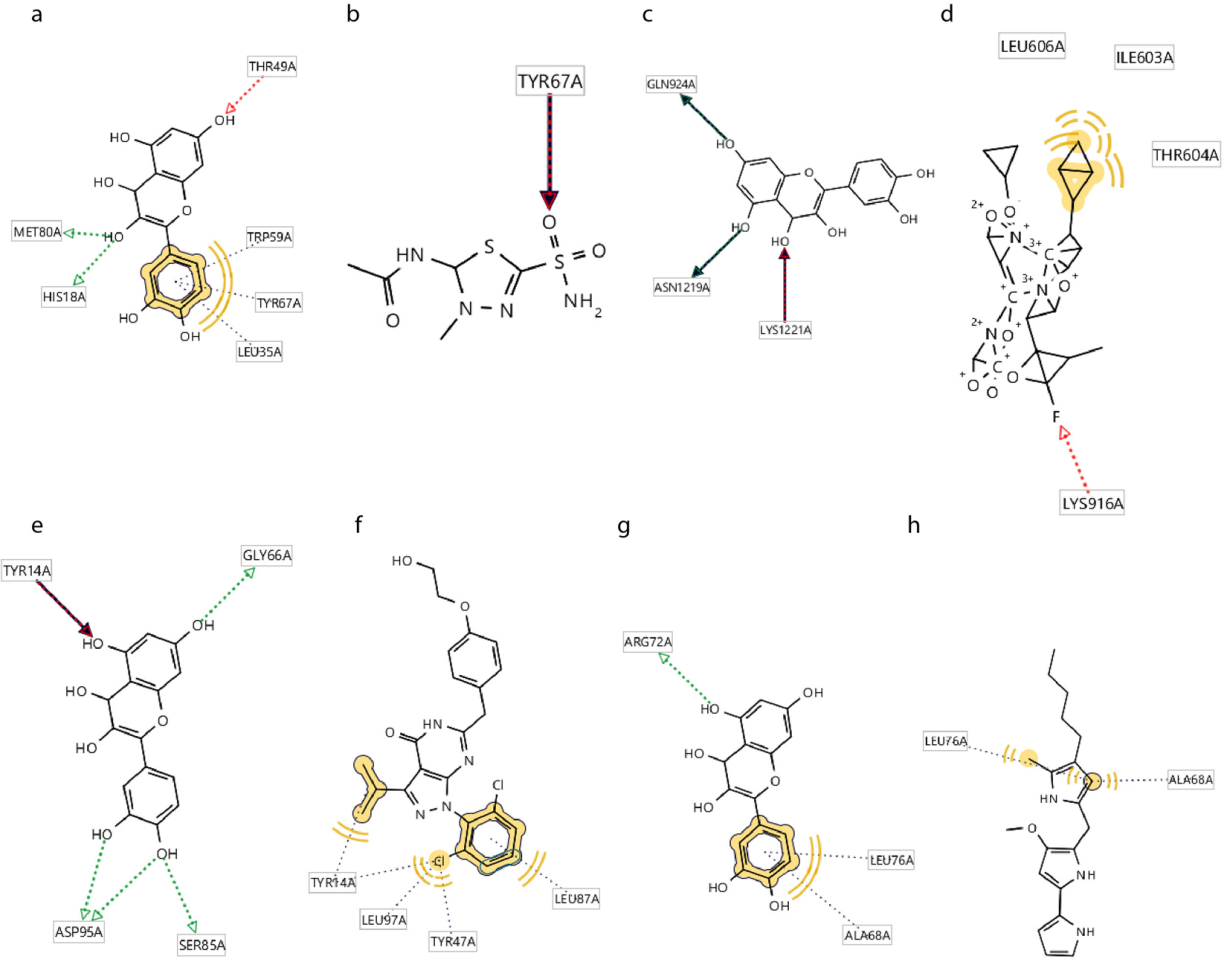
(**a**) CytC-Q, three hydrogen bonds and four hydrophobic interactions, (**b**) CytC-Inhibitor, one hydrophobic interaction, (**c**) Apaf1-Q, three hydrogen bonds, (**d**) Apaf1-Inhibitor, one hydrogen bond and two hydrophobic interactions, (**e**) CRK-Q, five hydrogen bonds and one hydrophobic bond, (**f**) CRK-Inhibitor, three hydrophobic interactions, (**g**) FADD-Q, one hydrogen bond and one hydrophobic interaction, (**h**) FADD-Inhibitor, two hydrophobic interactions.

Results obtained for CytC, Apaf1, CRK, and FADD which are key members of apoptosis pathway, also revealed that interaction between QT and CytC, Apaf1,CRK, and FADD include three hydrogen bonds (His18, Leu35, Thr49, Trp59, Tyr67, Met80 which all locate on CytC domain) (Fig. 8a), three hydrogen bonds and one hydrophobic interaction (Gln924, Asn1219, and Lys1221 all located on apoptotic protease activating factor domain) (Fig. 8c), five hydrogen bonds (Tyr14, Gly66, Ser85, and Asp95 locate on SH2 domain) (Fig. 8e), and one hydrogen bond and one hydrophobic interaction (Ala68, Arg72, Leu76 all located on DED domain of this protein) (Fig. 8g) respectively.

Furthermore, interactions of CytC, Apaf1, CRK, and FADD proteins with their inhibitors include one hydrogen bond (Tyr67 located on CytC) (Fig. 8b), one hydrogen bond and two hydrophobic bonds (Ile603, Thr604, Leu606, Lys916, allocated on apoptotic protease activating factor domain) (Fig. 8d), three hydrophobic interactions (Tyr14, Tyr47, Leu87, and Leu97 located pn SH2 domain) (Fig. 8e), and two hydrophobic interactions (Ala68, and leu76 located on DED domain of this protein) (Fig. 8h) respectively.

According to the previous studies, no relationship has been reported between these three proteins and LTP, while our results revealed a high interaction affinity between QT and these proteins specially FADD which has even lower Ki value than its specific inhibitor. Based on this finding, it can be suggested that the action mechanism of QT on the improvement of learning and memory depends not only on the maintenance of signal plasticity, but also is relied on the inhibition of the activity of proteins involved in the initiation and progression of apoptotic processes that in turn inhibit loss of neural cells and helps to maintain memory.

## Discussion

QT as an important flavonoid is found in vegetables and fruits, such as black and green tea and has potential in preventing cognitive inefficiencies at least in animal models^53–57^. There are contradictory findings of the beneficial effects of QT on learning and memory. Zhang and colleagues reported neuroprotective effects of quercetin on the reduction of oxidative stress and its hallmarks in subjects exposed to PM2.5^58^. Liaquat and colleagues found that QT prevents oxidative stress-induced memory impairments efficiently and improves memory retention^59^. In addition, Mehta reported a reduction of memory impairments caused by insulin resistance in rats by increasing the production of GLUT4 in the hippocampus^60^. Findings of Pei and colleagues also revealed that quercetin inhibits memory impairments via enhancement of Akt signaling pathway^61^. On the other hand, Dong and colleagues stated that quercetin enhances memory deficits in rats treated with D-galactose^62^. Similarly, Jung and colleagues demonstrated adverse effects of quercetin on learning and memory of normal mice via reduction of Akt and CREB proteins production^63^. Other groups of studies have discussed the inhibitory effects of QT. The effect of QT on the MAPK signaling pathway has been investigated, so that Warg *et al*., reported that QT has inhibited the liver fibrosis progression via inhibition of MAPK and inflammatory signaling pathways^64^. In addition, Kim and colleagues conducted a study on the effect of QT on cancer cell apoptosis through the MAPK signaling pathway. While they concluded that this signaling pathway and apoptosis are regulated through regulation of MSK1 and subsequent deacetylation of H3^65^. Studies conducted on QT and flavonoids and their interactions with proteins especially tyrosine kinase family have shown that flavonoids are able to inhibit tyrosine kinases via binding to the adenosine binding pocket and mimicking its binding^66^.

Nevertheless, poor solubility and the difficulty of QT to passage across the BBB restricts its effects in CNS^67,68^. A number of methods have been used to improve QT solubility by making water-soluble derivatives of QT^69^, using dimethylsulfoxide (DMSO)^70^, and complexation of it with cyclodextrin and liposome^71,72^. DMSO has vasoconstrictor and neurotoxicity effects^73^, and synthetic water-soluble derivative of QT has limited bioavailability^69^. Additionally, using cyclodextrin might have a nephrotoxic effect and liposome storage of QT may decrease its stability^74^. These limitations convince researchers to find a safe, constant, and efficient delivery approach to enhance the solubility and bioavailability of QT. The novel hybrid nanomaterials have been widely considered to overcome these limitations. Based on the results obtained from loading and release tests QT-SPION conjugate, synthesized by researchers in this study, is one of the newest delivery systems which increases blood circulation period of QT, in addition, to increase in the concentration of QT in the brain.

Kania *et al*. reported that liver and kidney are involved in SPION removal of the body in mice^75^. In this regard, it was reported that no significant toxicity has been observed using SPION in liver, kidney, and heart tissues^76–78^. In consistence with our results, no necrosis was observed in liver tissue of animals treated with SPION in the study of Silva and colleagues^79,80^. On the other hand, it has also been stated that NPs including SIOPN can pass through tissues including liver and be delivered to target organs^81^. In this regard, since we used higher concentrations of SPION and QT-SPION than mentioned studies, the presence of blue spots in Prussian blue staining and also significantly higher concentrations of iron on liver and kidney tissues can be due to higher administrated concentration. While higher concentrations of iron in the brain also revealed delivery of QT into brain tissues while no toxicity was observed inn fluorescent microscope pictures. Considering that no toxicity has been observed in studies using SPION in order to deliver drug compounds to tissues, more studies should be conducted in order to investigate tissue distribution of SPIONS.

In our recently published paper^44^ we discussed the factors such as synthesis method, size, surface coating, and conjugation procedure affect the pharmacokinetics and biodistribution of the SPIONs. In another paper, we showed that the SPIONs could enhance the amount of quercetin in both plasma and brain^45^. The results of this study showed that SPION acts as a carrier and increases the amount of quercetin in the brain tissue. The toxicity of SPIONs depends on many factors. Almost the same factors affecting pharmacokinetics and biodistribution of SPIONs affect SPIONs toxicity. Overall, the size, structure, shape, and coating have important effects on the cellular uptake, cytotoxicity, distribution, and clearance of SPIONs.

Once SPIONs enter to the gastrointestinal tract the epithelium uptakes them and SPIONs enter the systemic circulation. This is a useful approach particularly in the case of chronic diseases. SPIONs in our study absorbed in the gastrointestinal tract as we showed this previously^45^. In this study, we demonstrate the quercetin release from SPIONs, also the histological and paraclinical results showed SPIONs toxicity^45,82^.

There are many ambiguous points about the inhibitory effect of QT on proteins and its target proteins which mediate preventive effects, and also no or very rare studies on effects of QT on learning and memory of intact/healthy organisms, a bisectional study was performed. First, we examined the effects of QT and QT-SPION as a delivery system on learning and memory of intact/healthy rats.

The results obtained from the MWM of the current study showed that QT has a significant effect on improvement of learning and memory of intact Wistar rats that is inconsistent with previous experimental studies^16,83,84^. Significantly shorter escape latency during training days in QT (50 mg/kg and 100 mg/kg) and in QT-SPION (50 mg/kg) compared to the control group demonstrates improved cognitive functions in treated rats. These results are confirmed by the results obtained in probe trial day, in which QT-SPION (50 mg/kg) and QT (100 mg/kg) showed significantly more time spent in target zone and number of plate crosses compared to six remaining groups. Meanwhile, QT (50 mg/kg) did not show the desired results compared to QT-SPION (50 mg/kg) as we expected. Low bioavailability of quercetin has caused its insufficiency in the improvement of learning and memory as we also showed in bioavailability assessments previously^45^. However, contrary to our expectations, in comparison with the control group, QT-SPION (100 mg/kg) also did not show significant improvements in cognitive functions. According to the previous studies, SPION can interfere in the redox balance of the cells via Fenton reaction. Therefore, higher concentrations used in this treatment in conjugated form could probably be harmful to neurons that is mainly due to the accumulation of SPION in brain tissue^85–87^. So that, the use of SPION is not completely safe and it could cause cytotoxicity in higher dosages^44^. However, according to the results obtained by our research group, SPION caused liver toxicity in intact/healthy rats and cause an increase in MDA while neither QT nor QT-SPION groups (50 mg/kg and 100 mg/kg) didn’t show any change in levels of GSH, MDA levels, and CAT activity^82^. On the other hand, similar results were obtained for QT (100 mg/kg) and QT-SPION (50 mg/kg) through which effectiveness of manufactured conjugate can be confirmed. Regarding the results obtained from loading tests of QT-SPION in previous studies of our research groups as 42%, the concentration of quercetin in QT-SPION (50 mg/kg) is even lower than 50 mg/kg^45^. Based on these results, the use of QT-SPION removes the need for the use of high doses of QT because it increases the bioavailability of this natural compound.

Quercetin has been known as a very potent antioxidant flavonoid, but we have not used any oxidant agent in our treatments. Accordingly, the beneficial effects of quercetin and the designed delivery system are due to its potential for binding to proteins.

Studies conducted on QT and flavonoids and also their interactions with proteins especially tyrosine kinase family have shown that flavonoids are able to inhibit tyrosine kinases via binding to the adenosine binding pocket and mimicking its binding^66^.

Nevertheless, based on our searches, no general studies has been conducted on finding exact protein target to which quercetin binds. Since experimental studies were costly for our research group, and there was an interest in this issue, it was decided to perform an in-silico screening. In our study, RSK2, MSK1, Cdc42, and CRK have tyrosine kinase activity. Inhibition of Src kinase family also revealed that flavonoids bind to the tyrosine kinase domain of proteins^88^. However, interestingly MSK1-QT had lower interaction energy compared to MSK-1-inhibitor that is also inconsistent with our results. Despite the fact that inhibition of MSK1 and RSK2 leads to the survival of the cells and is an efficient intervention for treatment of several tumors and progressive diseases; however, inhibition of neural cell death is one of the most important mechanisms in order to prevent development of neurodegenerative diseases and also improvement of learning and memory which is directly associated to continuous production of transmitters and maintenance of LTP. Hence, it has been demonstrated that inhibition or downregulation of MSK1 and RSK2 is associated with the activation of signaling pathways related to the disruption of LTP and learning and memory^89^.

Besides, results obtained in a number of studies have revealed that upregulation of proteins involved in MAPK signaling pathway plays an important role in the phosphorylation of tau as the main hallmark of Alzheimer’s disease^90,91^. In contrast, studying the effect of mutation in MSK1/2 genes results in the reduction of proliferation of ischemic cells which can be considered as a treatment for this disorder^92^. According to the results obtained from these studies, inhibition of MSK1 and RSK2 as the key regulatory kinases of neural cell proliferation could be considered as a treatment in order to prevent the development of neurodegenerative diseases and stop the progression of these diseases, and most importantly to help maintenance of LTP.

Due to the role of cdc42 in preventing neurodegenerative diseases and also development and maintenance of LTP, overexpression of this protein has been suggested as a treatment for preventing neurodegeneration^93^. Yang and colleagues have shown that activation of Cdc42 depends on the structural changes which occur in the guanidine area exchange domain^94^. Therefore, blockade of this domain can be considered as a treatment to inhibit this protein. It has also been emphasized that regulation of Cdc42 is required in order to activate Cdc42 along with Rac protein in Wnt signaling^95^. Results of studying the signaling pathways related to the LTP have demonstrated that activation of Cdc42 as a target of activated MMP9 and 5-HT7R can lead to the dendritic remodeling which results in signaling plasticity and subsequently disruption of LTP while it may have a positive effect on reversal learning^96^. As a result, inhibition of Cdc42 may have two contradiction outcomes, one of which is inhibition of neural cell regeneration and the other one is LTP impairment. Considering that in the present study, QT and QT-SPION conjugate have led to the improvement of learning and memory in a dose-dependent manner, then it can be stated that QT can be a candidate regulator of Cdc42 in order to maintain LTP via regulation of Cdc42 in a dose-dependent mechanism so that doses higher than a certain range may not be effective or even have adverse effects.

P53 plays an important role in the regulation of synaptic plasticity via regulating the expression of genes involved in the LTP^97^. Besides, it has been demonstrated that expression levels of P53 increases in the hippocampus cells suffered from the deficiency of LTP. Consistent with our results, inhibition of p53 leads to the strengthen LTP and synaptic plasticity while also inhibits memory disruption^98^.

In contradiction, results obtained from memory impairment have revealed that treatment of LTP impairment cause recovery of P53 via inhibition of its oxidation^99^. P53 acetylation has also been introduced as an important stage in inhibition of progression of neurodegenerative diseases^100^.

According to our results and the proposed action mechanism by Hughes^101^ increase in cellular P53 levels leads to the cell apoptosis in neurons that results in neurodegeneration, therefore, inhibition of this protein may involve in inhibition of neurodegeneration^101^. Although, these results are consistent with our findings of the inhibitory effect of QT on P53, but since there was no applied injury in the neural cells, results have shown that P53 is important for maintenance of LTP, then it can be concluded that action mechanism of QT on improving learning and memory is not associated to inhibitory effect of QT on P53.

Interactions of QT have been investigated in various studies *in vitro* and *in vivo*. The results of a study revealed that interactions of QT with calcineurin restricts the interactions of this protein with its substrate resulting in the suppression of cytokine gene expression in spleen tissue of mouse^102^. An experimental study on QT inhibitory effect on the chloroplast ATPase complex revealed that QT inhibits hydrogen transfer and ATP production in chloroplast via interaction with CF and F0^103^. The interaction energy of QT and α-glucosidase of S. cerevisiae is less than this energy between QT derivatives and the same protein, then it is suggested that similar studies with the present study could be conducted using bioinformatics softwares^61^. Interactions of QT with albumin and ovalbumin have been reported in several studies^104^. QT has been suggested as an inhibitor for arginine kinase in insects in which it interacts with Trp amino acid located in the active site of this enzyme^105^. Consistent with our study, researches on therapeutic effects of flavonoids including QT on neurological impairment revealed that QT forms a stable complex with IR3, 2PRG with interaction energies within the range of 7–8 kCal/mol using *AutoDock* software and these energies have been considered as desirable compared to the standard international energy^106^. The anti-inflammatory effect of QT has also been investigated through studying the interactions of this flavonoid with Cox-2 and obtained results demonstrated the high dock score due to two hydrogen bonds with amino acids in active site of this proteins; however, the obtained scores haven’t been compared with other known inhibitors of this protein^107^. Studies on the effect of QT on learning and memory or neural damages have shown that QT is a very effective inhibitor in in-silico predictions.

Lockshmi and colleagues evaluated interaction energies of 7-Oβ-D-glucotryanoside with Akt1 while an appropriate inhibitory effect was predicted and this finding was confirmed via reduction of the expression level of apoptotic proteins^108^. Nevertheless, no comparison was conducted with other inhibitors of this protein.

The inhibitory potential of QT has also been investigated on IKKβ as an important member of inflammatory pathways which has also been studied in the mentioned study while similar to the other studies, the obtained results were not compared with a therapeutic inhibitor of this protein^109^.

Docking analysis of QT and flavonoids has also revealed that QT forms more stable interactions with tyrosine in active sites of kinases of Src family through the formation of several hydrogen bonds which is consistent with our results^88^. Since induced activity of acetylcholine esterase (AchE) has been shown to be a hallmark of Alzheimer’s diseases as a prevalent neurodegenerative disease, the inhibitory effect of QT and a number of flavonoids was studied against this enzyme and a high potent was predicted for interaction with this enzyme which was also confirmed in the subsequent studies^110^. Similarly, results have been obtained in *in vitro* and *in vivo* studies on memory impairment have demonstrated that QT and flavonoids exhibit desirable inhibitory effects on AchE and therefore can be considered as a treatment for AD^111^.

All of the results have also been produced using Q-SAR software and studying the potential of QT to inhibit AchE in 2013 in which QT binding affinity (−8.8 kCal/mol) was compared with AD drug, Deponzil (−7.9 kCal/mol) which was due to the higher numbers of hydrogen bonds than the drug which all are consistent with results of our study demonstrating higher binding affinity of QT than proposed drugs and forming more hydrogen bonds^112^. However, our study is the first study as a comprehensive comparative evaluation of the action mechanism of QT on the improvement of learning and memory via interfering LTP, neurotrophin, and apoptosis signaling pathways in intact rats.

Considering that our results have been obtained from intact rats with a normal lifetime and also due to the results obtained from MWM demonstrating improvement of spatial learning and memory in the groups of rats treated with QT and QT-SPION compared to the control groups (control, sham, and SPION groups), therefore it can be suggested that QT in free and conjugated forms and especially QT-SPION form improves learning and memory via regulation of key kinases involved in neurotrophic, apoptosis, and LTP signaling pathways, and hence it can be considered as a candidate treatment to prevent development of neurodegenerative diseases in addition to improving learning and memory. Noteworthy, QT complex with a number of the proteins including MSK1, RSK2, P53, Cdc42, CytC, FADD, APAf1, and CRK (MSK1-Q: 94.82, MSK1-inhibitor: 74.61; RSK2-Q:31.97, RSK2-Inhibitor: 8.52; P53-Q: 32.77, P53-Inhibitor:28.08; CytC-Q: 59.24, CytC-Inhibitor: 46.44; Apaf1-Q:443.27, Apaf1-Inhibitor:426.47) had higher Ki values than specific inhibitors of these proteins; however Ki-values of QT interacting with Cdc42, FADD, CRK (Cdc42-Q:141.32, Cdc42-Inhibitor:141.63; FADD-Q: 13.42, FADD-Inhibitor:13.63; CRK-Q:15.09, CRK-Inhibitor: 22.25) was lower than the inhibitors of these proteins. Ki value is an indicator of inhibitor determining its effective concentration in which half of the protein activity is inhibited. In addition, low compatibility of QT is a barrier against the effectiveness of this valuable compound that leads to its degradation in a few hours after administration and its effects become non- significant, in contrast, enhancing the bioavailability of QT using conjugated nanostructures such as Fe_3_O_4_ is a novel approach in order to use all the beneficial effects of QT. As it was observed in our study, enhancement of QT via QT-SPION conjugate led to significant improvement of learning and memory in comparison with the group treated with free QT. In-silico studies conducted to evaluate interactions of QT and proteins that mainly have been introduced as the potential targets of QT in the experimental studies. Comparison of the binding energies of QT to glycogen phosphorylase with gallic acid and metformin showed that QT binds to the target protein with a lower binding energy; however, no comparison was conducted with the specific inhibitor of glycogen phosphorylase^113^.

As a conclusion, QT can be considered as a novel inhibitor of proteins involved in MAPK and apoptosis signaling pathways by interfering the main activity domains of proteins and can result in the maintenance of learning and memory via strengthening signal plasticity and LTP.

However, since the required concentrations of QT is higher than specific inhibitors, and the use of conjugated nanoparticles led to obtaining better results in learning and memory of healthy rats then it can be proposed that QT-SPION nano-conjugate even at lower concentrations than QT can lead to better results due to the bioavailability and circulation time enhancement of QT in the blood flow that leads to a higher antioxidant activity and more efficient interactions of QT with proteins.

The main limitation of the present study is the lack of experimental studies on interactions of QT and proteins involved in learning and memory. However, we tried to simulate all potential interactions of QT and proteins in rats which were young and had no memory impairment. For this purpose, we collected PDB formats of proteins obligated to rat and studied their interactions with QT. However, an experimental screening is also required in order to complete our findings and find new targets for the treatment of diseases using QT. We hope these primary findings will help other researchers even for investigating the inhibitory effect of QT on any of proteins in details.

### Experimental procedures

#### QT- SPION nanoparticle preparation and characterization

QT was purchased from Sigma – Aldrich chemical (Sigma–Aldrich Co., St. Louis, MO) in an anhydrous powdered form. The protocol of QT-SPIONs preparation was in accordance with a previous report^50^, but with certain modifications. Briefly, the chemical co-precipitation (CPT) method was used to synthesize dextran coated SPIONs. Anhydrous FeCl_3_ (3 mmol, 0.48 g), FeCl_2_ (1.5 mmol,0.29 g) and dextran T-10 [50 ml, 0.5% (w/w)] were dissolved in deionized (DI) water, were mixed and then were put into a three-neck flask equipped with a mechanical stirrer. In the following, the ammonia solution was added into the mixture, until the pH of the solution reached to 9. This solution was kept at 90 °C for 2hs with constant stirring, and then the resultant precipitate was collected using a strong external magnet. The supernatant was washed several times with DI water and ethanol and was dried in an oven at 70 °C overnight. In order to make QT conjugated magnetite nanoparticles (QT-SPION), QT was added to dextran coated SPIONs via EDC/NHS protocol. For this purpose, 100 mg of dextran coated magnetite nanoparticles, NHS (6 mg) and EDC (4 mg) were sonicated in 100 ml Acetate Buffer (0.1 M, pH 5.0) and DI water for 15 min. Then, the resultant precipitate was added to quercetin (100 mg) and was dissolved in 100 ml of DMSO and stirred for overnight at room temperature. Next, quercetin conjugated magnetite nanoparticles were isolated from suspension via external permanent magnet and washed several times with DMSO, DI water and acetone and finally dried using freeze drier. The synthesized QT-SPIONs were characterized by various analytical techniques.

The FT IR spectroscopy was recorded on a Jasco 6300 spectrophotometer in transmission mode with KBr pellets, operating from the wave numbers between 400 and 4000 cm^−1^. XRD patterns of magnetite nanoparticles were collected using Cu K1 (k = 1.54056 Å) radiation on a PANalytical X’PERT PRO powder X-ray diffractometer at the room temperature. The morphological features were obtained on a Hitachi S–4700 field emission – scanning electron microscope (FE–SEM), equipped with an energy dispersive X-ray analysis (EDAX) detector. The particle size distribution of the prepared magnetic nanoparticles was determined by dynamic light scattering (DLS) (VASCO Instruments, FRANCE, CORDOUAN TECHNOLOGIES).

#### Determination of quercetin loading

Quercetin loading of QT-SPIONs was determined by UV–Visible spectrophotometer at 377 nm. For this purpose, 1 mg of quercetin conjugated magnetite nanoparticle was dissolved in 10 ml PBS and centrifuged at 8,000 rpm for 30 min. The drug loading was found to be 42% calculated by the following equation:

$$Drug\,loading\,( \% w/{\rm{w}})=\frac{Mass\,of\,drug\,loaded\,in\,NPs\times 100}{Mass\,of\,nanoparticls}$$

#### Animals

Adult male Wistar rats, weighing 180–230 g were purchased from Royan Institute (Isfahan, Iran). The animals were randomly divided into eight groups and were housed 3 to 5 animals per cage at a temperature of 25 ± 2 °C and a 12-h light/dark cycle (lights on at 07:00 a.m.). All rats were maintained in the laboratory for two weeks in order to adapt with the environment while unlimited food and water were provided for them and the floors of the cages were covered with wood shavings, which were replaced every 3 days. The state committee on animal ethics, Shiraz University, Shiraz, Iran, approved the experiment protocol (IACUC no: 4687/63). Furthermore, all experiments were carried out in accordance with the guide for the care and use of laboratory animals (USA National Institute of Health Publication No. 80–23, revised 1996) and were reviewed and approved by the animal ethics committee of the University of Isfahan.

For oral administration, QT, SPIONs (in powder form), and QT-SPION (in powder form) were suspended in deionized water (DI) immediately before administration. Dispersions were kept on shakers during the experiments in order to prevent probable agglomeration of quercetin and nanoparticles.

Animals were randomly divided into eight groups including six rats each.

Group 1: Rats in the control group didn’t receive any drug or vehicle.

Group 2: Rats in the sham group were treated with vehicle of QT (dispersed in deionized water (DI)) for seven days.

Group 3: Rats were treated with 50 mg/kg/day of free QT (dispersed in distilled water) for 7 days.

Group 4: Rats were treated with 100 mg/kg/day free QT (dispersed in distilled water) for 7 days.

Group 5: Rats were treated with 50 mg/kg/day SPIONs (dispersed in distilled water) for 7 days.

Group 6: Rats were administered 100 mg/kg/day SPIONs (dispersed in distilled water) for 7 days.

Group 7: Rats were administered 50 mg/kg/day QT-SPIONs (dispersed in distilled water) for 7 days.

Group 8: Rats were administered with 100 mg/kg/day QT-SPIONs (dispersed in distilled water) for 7 days.

All formulations were given orally at a daily dose for a period of seven days. The behavioral assessment was carried out during the treatment method.

#### Morris water maze (MWM)

**Figure 9:**
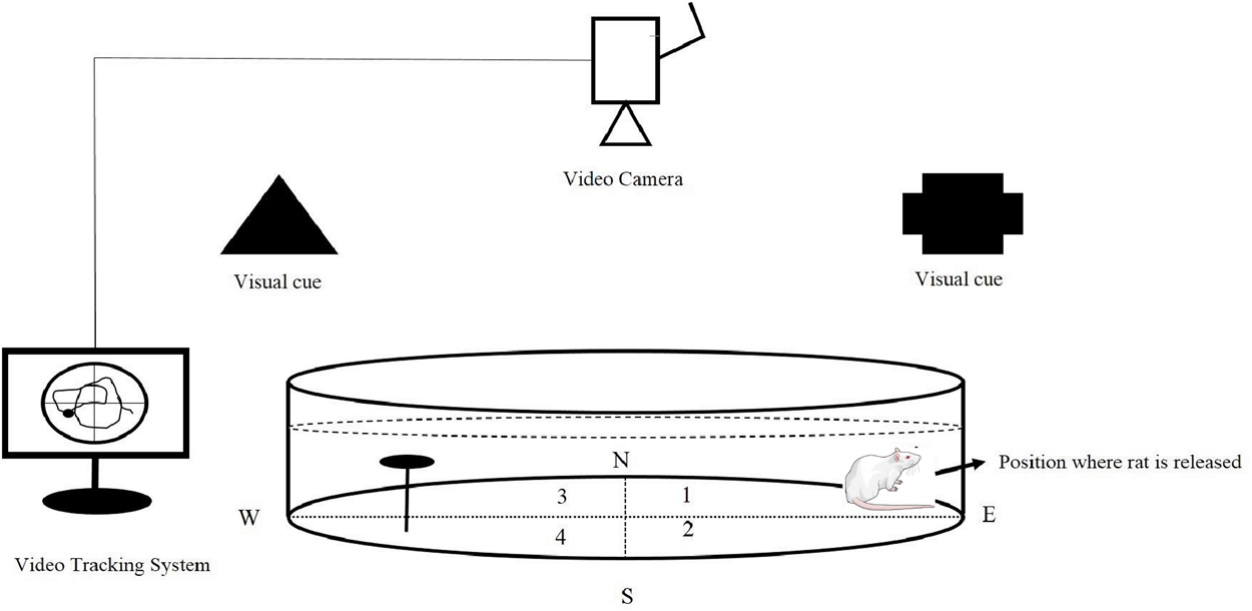
Schematic illustration of Morris water maze (MWM). The pool was divided into four quadrants (N, north; S, south; E, east, W, west). A platform with 8 cm diameter was placed one cm below the water level in the middle of one of the quadrants as the hidden goal platform. The rat swimming from the start point to the goal was tracked by a video camera connected to a video tracking system. Image of rat from Mind the Graph (https://mindthegraph.com/) under a CC BY SA 4.0 licence (https://creativecommons.org/licenses/by-sa/4.0/).

The spatial memory was examined using a *Morris Water Maze* (*BorjSanat*, *Tehran*, *Iran*) according to the previous procedures^114^. Rats were trained to swim to reach a hidden platform in a circular pool (180 cm diameter, 60 cm height,) located in a darkened test room. Escape latency, moved distance, time spent in quadrants and number of platform crossing were measured as indicators of learning and memory using a video camera, mounted in the ceiling above the center of the pool, and a computerized tracking system (VideoTracking Software, designed by BorjSanat Company). The pool was filled with water (23 ± 2 °C) to a depth of 50 cm. Four equally spaced points around the edge of the pool were designed as N (North), E (East), S (South) and W (West). A colorless round platform with 8 cm diameter was placed one cm below the surface of the water in a fixed position in the middle of the NE quadrant of the pool (Which will be called as zone 3, Fig. 9). The water was colored with powder milk to hide the location of the submerged platform. All rats were assessed using *MWM* for 5 consecutive days from the second day of treatment. Each rat was trained for four turns per day.

In each turn, the rat was released in one of four quadrants facing the pool wall so that at the end of the training day, all rats had been released in all quadrants once. Every training last for 60 seconds. Rats continued to swimming until they could find the platform in Zone 3. If they couldn’t find the platform, experimenter conducted them through the platform and let them stay on it for 15 seconds. The next round of training was started only when all of the rats were trained in the present round. Criteria were recorded: escape latency as latency to find the hidden platform at trial days, path length as the distance moved by rats during each training turn.

As the final step, on the sixth day, the platform was removed and the probe trial was implemented in order to evaluate the reference memory. Two criteria recorded: time spent in the target zone in which the platform was placed during training days and plate cross as times rats cross above the position of the probe at probe trial day when the hidden platform was removed.

#### Histological analysis and iron determination

Histological studies were performed in order to determine SPION tissue distribution. First, organs of examined rats including brain, liver, and kidney were separated and stored in 10% neutral buffer formalin for 24hs. Then, tissues were embedded in paraffin and blocks were used to cut thin layers of tissues. Tissue sections were first stained using hematoxylin and eosin and then stained using Perl’s Prussian blue. Prepared samples were then photographed using a fluorescent microscope (IndiaMart).

In order to determine concentration of iron in tissues, inductively coupled plasma mass spectrometry (ICP-MS) was used. Segments of brain, liver, and kidney tissues at the same weight were prepared. In the following, they were treated with HNO_3_ (65%) for 16 hours in room temperature and were filtered until a transparent solution was obtained. Samples were assessed using ICP-MS. In this technique, liquid samples were converted into aerosol form. The aerosol formed samples were decomposed via a radio frequency field under a very high pressure, generated using Argon. Ions were extracted and sent into detector. ICP-MS is commonly used to measure concentrations of metals.

### Computational methods

#### Preparation of protein structures for docking calculations

All proteins involved in the neurotrophin signaling pathway and related signaling pathways of apoptosis were entered in a database of this research using KEGG database (http://www.kegg.jp/keggbin/highlight_pathway?scale=1.0&map=map04722&keyword=neurotrophin). Then the entry numbers presented for proteins in the KEGG database were used to search the proteins in the *Protein Data Bank* (*PDB*) (http://www.rcsb.org/pdb/home/). *PDB* files of all proteins were studied after removal of water molecules and other molecules and then the structures of pure proteins were saved in the pdb format using *Molegro Molecular Viewer* (*MMV*) software. The list of proteins has been presented in Table 1. Additionally, the structure of QT in sdf format was obtained from (https://pubchem.ncbi.nlm.nih.gov/) with the *PubChem* CID of 5280343, the molecular formula of C_15_H_10_O_7_, and molecular weight of 302.238 g/mol (Fig. 1). The sdf files of inhibitors of each protein were also obtained from *PubChem* after searching in *PubMed*, *ScienceDirect*, and *PubChem*.

All the obtained structures including proteins and the ligands were then analyzed using the *LigandScout* software in order to minimize the energy of structures before obtaining the docking product using MMFF94 force field^115^.

### Docking calculations

#### Hex

Hex docking software was used in order to implement docking procedures of QT with proteins of the signaling pathways including apoptosis, and neurotrophin signaling pathway. *Hex* software has been developed in order to calculate the minimum free energy obtained from the best interaction mode through the analysis of various interaction modes during the lowest possible time through the Fast Fourier Transform (FFT) correlation technique. This software assumes ligands rigid and can calculate about 1000 docking predictions in each docking process^116^. The parameters set for this purpose include:

Correlation type: Shape Only; FFT Mode: 3D; Grid Dimension: 0.6; Receptor range: 180; Ligand Range: 180; Twist Range: 360; Distance Range: 40; Translation step: 0.8; Box Size: 15; Solutions: 2000.

Additionally, the proteins which had no pdb query for the complete structure were not enrolled in the database.

#### Auto Dock

*AutoDock* is a docking program used for calculation of the interactions between proteins and ligands using and the binding energy using empirical free energy force field with a Lamarckian Genetic Algorithm. *AutoDock* 4.2.6 considers receptor protein and ligand as relatively flexible and evaluates several interaction states with the side chains which are selected by using and displaying the best state as output^117,118^. In the present study, the grid was selected so that all the possible interactions of ligands were calculated and the best state was obtained. This is a time-consuming step.

#### Ligand Scout

*LigandScout* is a pharmacophore identifying software which evaluates interactions of proteins and ligands in terms of the interactions formed between ligand and side chains of proteins and provides information on hydrogen bond acceptor and donor groups, displaying the lipophilic area, aromatic and charge transfer interactions^119^. This helps the user to identify the interactions and find the required changes in ligand in order to design more efficient inhibitors. In the present study, we used the features of *Ligandscout* in order to find the interactions of QT with proteins and compare the interactions of inhibitors with the proteins.

### Statistical analysis

The results were reported as mean ± SEM for the six independent experiments. The one-way ANOVA followed by a tukey,s multiple comparison tests, two-way ANOVA or paired t-test were used to estimate the difference as multiple comparison tests (*GraphPad Prism* software version 6). P < 0.05 was considered to be statistically significant.
